# The metaverse of violence

**DOI:** 10.3389/fsoc.2024.1147627

**Published:** 2024-04-23

**Authors:** Michelangelo Pascali

**Affiliations:** Department of Social Sciences, University of Naples Federico II, Naples, Italy

**Keywords:** metaverse, violence, law, rights, crime, deviance, physicality, virtuality

## Abstract

The metaverse appears to be a composite concept and a complex environment from an ontological perspective and from a purely dimensional point of view. Exploring its defining features not only allows one to identify the nature and effects of the social relations existing therein, but also influences the legal reading of what it contains and produces. Bringing to light the peculiar characteristics of the metaverse—for which the dichotomy between “real” and “virtual” sounds outdated—this article emphasizes the urgency to rethink the traditional forms of interpretation and design of preventive and repressive measures to counter deviant and illegal phenomena of a violent nature.

## 1 “Social territories”: representations and essence of the metaverse

Space constitutes one of the dimensions through which human action unfolds. The concatenation of territories and events takes on peculiar declinations that sink their roots and explain their effects in the complex realms of Law, where the totality of social relations—at the same time an elusive reality and a stable model—is conceived and systematized. Given that “there is no space independent of subjects” (von Uexküll, 2013, p. 75), it is precisely in the manifold, and not always peaceful, relationship between the environment and its users that aspirations and limits of human projections are felt, which largely lead to visions and re-visions of the history and future of rights. This applies not only to genuinely physical spaces but also to digital spaces and even to the combination between them.

The emergence of the metaverse, understood as a universal environment capable of forging and intercepting the complexity of the network of social connections in a new, typical digital extension, appears to be an event, albeit with non-homogeneous and non-linear features, with the potential to disruptively affect our understanding of reality. In particular, the innovation resulting from the appearance of the metaverse cannot be reduced to a process of mere transposition of a concrete, non-digital reality carried out by means of a next-generation “internetization.” On the contrary, it should be taken as one step on the path toward the establishment of a stand-alone reality whose essence lies in the lack of separation between the spheres of existence.

If depicted as the three-dimensional cyberspace in which natural persons can operate, produce, and interact in different ways for a variety of purposes, and by making use of increasingly customized tools and services, the digital universe thus constituted—endowed with persistence and interactivity among interconnected contexts enabling real-time actions and relations to take place (Ball, 2022)—ultimately molds a reality that is not fictitious (any longer), but if anything is implemented. Accordingly, such a technologically affirmed reality, made up of informational galaxies or suburbs, is not conceivable as a parallel dimension but rather as part of mutually integrated realities.

At the heart of its development, there is an acceleration in scientific applications, an advancement in the branch of digital ecology, and, finally, a technological integration of “virtual” and “physical” contents (China Institutes of Contemporary International Relations – CICIR, 2021), through that path of hyper-connection that has already largely hybridized the frontiers of an “onlife” life (Floridi, 2015)—commonly referred to with the term *phygital* (Council of the European Union, General Secretariat – C.EU, 2022).

Nonetheless, it is still unclear whether such a scientific growth will contribute to the consolidation of greater forms of “technological anthropocentrism” (Dertouzos, 2001), also considering that the nature of the *prosumer* remains controversial in terms of economic and commercial rights—being in unstable equilibrium between freedom and imposition (Pascali, 2022).^1^ Nevertheless, the user of this new world shows a remarkable potential to become a driver of innovation—at once consumer and producer of ideas—and, as such, to generate an inevitable, albeit not total, horizontality of contents.

Rejecting the assumption of the existence of an absolute dualism between what is real and what is virtual thus becomes pivotal to a proper understanding of the theoretical frameworks within which the socio-digital objects of research are to be discussed (Rogers, 2013; Caliandro and Gandini, 2017). Accordingly, the methods used to investigate “digitality” must conform to the permeability of the material and digital spheres, taking into account the possibility of using them as analysis data (and “pre-data”) that can be acquired. The underlying idea is, indeed, that the “boundless” virtual space and the concrete reality (*cf.* Stephenson, 1992) are not separate dimensions (although subject to a mutual and permanent exchange, *cf.* Riva, 2014, p. 60), but concurrent spheres that might experience overlaps and fusions and sometimes reflect different aspects of the same reality.

In an epistemological and methodological perspective, both dimensions are thus to be regarded as “real” since they have a *real* impact on phenomena. In other words, the notion of the circularity of the spheres in which a given phenomenon occurs is preliminary to any study of the phenomenon itself. This also implies that even in cases where the event seems destined to be confined to a single, specific dimension, it could progressively and massively explode in such an overall horizon and finally deflagrate in circumscribed spaces.^2^ Based on the above, neither concrete reality nor cyberspace should be qualified as “abstract,” especially considering the socio-psychological implications of what can be achieved in the metaverse (Oleksy et al., 2022).

To clarify the definition of “virtual/digital,” it is important to note that the incorporation of new technologies into social worlds requires a re-discussion of the concept of individuality (Lupton, 2015, p. 188 ff.), as well as considering the ways in which social research may be “reinvented.” This is particularly relevant given the purely social character of these technologies themselves (Marres, 2017). By observing how actions and subjects interact cohesively in a diverse environment, we can gain a better understanding of critical analysis perspectives (Orton-Johnson and Prior, 2023) and the need to reassess the value of spatial boundaries that have traditionally been considered fixed.

The growing weld between “*meatspace*” and *“cyberspace*” (Weatherby, 2018) has thus been established for defining purposes. Furthermore, space—which is no longer conceived merely as physical data but also as a social construct—now appears to be inevitably thinkable in terms of its becoming, also through the transduction process by means of which software produces its conditions of existence, in an endless generative work that goes beyond a simple mixing effect (Accoto, 2017, p. 88 ff.). Then again, techno-regulation, in itself, is evidently readable as a process capable of conforming the environment in which subjects interact (Durante, 2019, p. 49).

## 2 Dimensions of violence

The digital dimension, which is now included in almost all areas of social relations, must also necessarily be taken into account by studies that deal sociologically with the phenomena of deviance and crime. This entails the observation of new social facts that can be included in these macro-categories and obliges us to address, first and foremost, novel definitional problems.

With regard to the “original” character of digital phenomena of this origin and of a “violent” nature or purpose, it should therefore be noted that even many of them are not categorized in a real and a virtual level, nor is there exclusively a synergy between sectors that are in any case (thought and acted upon) as distinct. Rather, in their diversity, these episodes subsist and move in a substantial-conceptual unicum.

It should then be noted that the novelty present and to be considered is both social and legal, even from a socio-legal perspective. It is precisely the criterion of novelty that can be used to detect the specific nature of the matter under consideration, being expressed with respect to many of the aspects in question. More precisely, the innovative part may, indeed, from time to time, concern the range of means, the type of actions, the actors involved in these dynamics, the place where they are expressed, and even their very purpose.

According to an approach that largely assumes a divergence—at least an ideal one—between areas, a distinctive criterion that can be used to assess the ontology of the subject at hand could be to ascertain whether the acts examined are completely original digital-real expressions or new digital manifestations of classic real dynamics.^3^ Virtual and physical environments should thus be considered as speculatively endowed with exclusive meanings and bearers of exact terms, while maintaining their mutual accessibility. In this way, the digital domain becomes the expressive and instrumental space of actions,^4^ as in the second case, or otherwise precisely the source and reason for those actions (thus relating to an activity of production and not only of reproduction, and even less of pure representation; Pascali, 2008). In this sense, it is still possible for identities, whether virtual or digital, to be dissociated, in part due to online disinhibition effects, which are linked to processes such as dissociative anonymity, invisibility, asynchrony, solipsistic introjection, dissociative imagination, and the minimization of authority (Suler, 2004). Thanks to the synchronism and immersiveness afforded by technologically mediated actions, where the very perception of the existence of the tool employed disappears, what is accomplished^5^ can be influenced by one’s own digital representations (according to what has been termed the “Proteus effect”; Yee et al., 2009). Noting the above has led to highlighting the “normality” of online behavior connected to processes of de-individuation, with obvious repercussions on the physical plane as well. The danger of unknowingly engendering widespread body dysmorphism must therefore be set alongside that of deliberately generating sensitive dysfunctions in others.

Nevertheless, in a legal interpretation of any unlawful acts of a violent nature attempted or carried out in the metaverse, the consideration of a basic uniqueness (or lack thereof) in connection with the *locus commissi delicti* and/or the place where the effect of the offense may have occurred leads, in any case, back to the proper scope where the typical constituent elements of criminal action can be included. At the outset, a crucial consideration is the classification of the metaverse from a spatial standpoint,^6^ as it has also been characterized as a heterotopia rather than a place (De Simone, 2023). This has inevitably impacted the definition of the criteria for the identification of the process underlying the actions performed.

Instances of cyberbullying, extortion with sexual motivations or characteristics, unlawful media dissemination of other people’s data and images, including intimate ones, and cyber instigation to perform unlawful or self-harm acts—all of which, in a broad sense, have underlying psychic dynamics of coercion and physical or moral violence—as well as clearly demonstrating the real repercussions of virtual environments (even constituting hypotheses of death as a consequence of another crime), already widely established, require referral to and comparison with hypothetically incriminating offenses, either generically or specifically envisaged.

For other legal profiles, it should be noted that law enforcement interest in the metaverse^7^ is now apparent (Interpol, 2022a),^8^ not only as a possible area of attention and subsequent repressive action but indeed as a site where to establish a *normal* presence and also for ordinary prevention (Ferri, 2022). In fact, the metaverse seems to be already characterized by a high degree of harassment and similar behaviors (Patel, 2021). Moreover, such behaviors are destined to result in increasingly invasive effects thanks to improvements in haptic technologies, which can bring about a shift from the sense of pleasure to the actual sensation, not merely psychological, of the violence endured (Mazer, 2022). To address this problem, efforts are being made from several fronts and for different reasons. In addition to the necessary, but sensitive, delegation of the issue to “experts” on the matter, this also points back to the question of risk management, implemented alternatively or in parallel by private companies that organize metaversal information and social spaces. These are obviously affected by business logic (Lombardi, 2022; Pierattini, 2022), with potential improper intrusions in terms of freedom rights. Indeed, it is precisely the issue of governance of social conflicts—together with that of recognizing the nature and property value of the new *res* created (and, therefore, their necessary protection; Wildman and McDonnell, 2020)—that appears to be central already in the first articulated configurations of “virtual worlds” (Lastowka and Hunter, 2004), to be included in a complex geopolitical framework (*cf.* Vanorio, 2021).^9^ This theme brings us back to the question of examining the legitimacy of the rules imposed in virtual communities. For some (Rolfes, 2022), such legitimacy could be positively qualified only through the prerequisites of consent by the individual user and pursuit of the common good of the user community, which, moreover, must be filled with meaning.

Thus, there is the rise of new modes and new forms of “abusive” behavior in a system that leads to the realization of that pre-conceived perception of an unmediated experiential presence (unmediated but direct and “natural,” as well as immersive, shared, and dense with a realism that is also social; Lombard and Ditton, 1997). The problem represented by such a rise is logically followed by the central question of the anticipatory definition of those behaviors, and that of the legitimacy and appropriateness of the remedies put in place to prevent or repress them.

Alongside this, there remains the discussion on the legal liability profiles to be recognized for the various activities carried out by providers of telematic services (Fornasari, 2004, p. 423 ff.), which cannot be limited to omissive conduct (Bassoli, 2009, p. 170 ff.). Therefore, it is important to consider the legal and administrative role of digital platforms in contemporary society. Despite the common narrative (*cf.* Morozov, 2011), these platforms may actually encourage illicit and deviant behavior (Pascali, 2023) and its public dissemination through their affordances. Additionally, the restructuring and reorganizing process of the underlying network technologies can have an impact on the architecture of relevant publics (Boyd, 2010). Furthermore, this is related to a broader question about the possible and desirable qualities of an “electronic” justice system (Kostenko, 2022), especially given significant scientific advancements (Scorza, 2022, p. 13).

In a way, we are witnessing an incessant chase between new socio-technological developments and rights that can be invoked, also in relation to concepts such as personal identity^10^ and social control and to processes of behavioral engineering, with which every “metacosm” is interwoven (*cf.* De Vivo, 2009).

For all these aspects, we are brought back to the debate on the nature of objects and events present in these realities, between “realist” and “irrealist” positions, with all the nuances that can be extended therein (McDonnell and Wildman, 2019) and the applicable distinctions between digital and virtual goods.

Incidentally, it must be said that even for these matters—which refer, moreover, to a general jurisdictional question—one cannot overlook how the all-encompassing centrality of digital forms of expression during the pandemic confinement have certainly contributed to accelerating the embryonic establishment of models of contrast.

## 3 Legal issues: discussions and concluding remarks

The diminishing of a rigid reading of illicit acts carried out through the use of new technologies as purely “projective” manifestations of a traditionally concrete action seems appropriate for an exact framing of “computerized/realized” deviant and criminal behavior of a violent kind—linked to conditions, intentions, and actions—which give different form to the already detected processes of victimization occurring in online spaces (Martellozzo and Jane, 2019).

The assumption of this non-disjunctional action must therefore be compared with the hypotheses of the separation of rights and those rights themselves.

In addition to the inherent long-standing investigative difficulties, for instance, pertaining to the existing mechanisms for identifying in the real world those who operate in the virtual world, through and behind screens (with repercussions, therefore, also on the judicial formation of evidence; Aterno, 2023, pp. 428 ff.), the prosecution of crimes belonging to the above-mentioned categories raises complex issues. Here, it is necessary to relate to a more general discourse on the hypothetical inadequacy of the schemes that govern criminal offenses and, at the same time, to possible reform proposals in light of the protections underpinning it.^11^

On the one hand, a clear penal caesura between the plans and subjective boundaries of action, though logically clear and for many aspects obligatory, risks being the premise of operations that are unsuitable for fully containing cases that are “elusive” from this point of view. On the other hand, regardless of the hypothesis of an effective theoretical-paraxial deconstruction of the liberal paradigm in the face of new actions proper to *homo communicans* who came out of the networks of interplanetary communication (Torrão, 2014, p. 61), the possible extension of the criminal prosecution plan, without a decisive framing within the constituent cornerstone of the field, appears to be a prospect with extremely serious consequences and, even more so, a systemically unthinkable one. Although some pronouncements on the subject of the recognition for legal purposes of the character of a public place or a place open to the public with regard to the immateriality of virtual squares (*cf.* Criminal Court of Cassation, sect. I, sentence no. 37596/2014, on the subject of harassment or disturbance of others) appear to be endowed with a certain exegetic consistency, the risk is still that of an improper and anti-systemic use of the existing cases “for the (social) good.”

In any case, as far as the subject of criminal jurisdiction is concerned, it is necessary to refer to the criterion of maximum attractiveness of the sanctioning instrument for the Italian legal system—even in the face of the complex dynamics of decentralization and, disregarding other parameters,^12^ whereby, *inter alia*, the unlawful act is judicially prosecutable even when performed by a foreign citizen and through platforms with a foreign registered office that have an effect on Italian soil.^13^ Nevertheless, in the interpretative adaptation of the forecasts and objections from the physical reality to the digital sphere, we do not refer only to the question of the identification of the spatial location of the acts or the results of the offense and not even to the classical discourse on the means of the offense, but to a differently genetic and real profile. In some respects, the operational and promotional aspects of violence acted out in this manner should, first and foremost, be seen as firmly materializing (or not materializing) a constitutive factor of existing offenses.

Should one then assume—for the sake of argument—that in the cases under evaluation one does not have (a centrality of) augmented reality or mixed reality but speaks exclusively of virtual reality, acting through simulations having the characteristics of reality, and, in this, the metaverse thus becomes a conceptually independent world system, but coexists and interacts in real time with the realm of physicality? This coexistence and simultaneity of physical-virtual realities also pose the problem of the dimension of subjectivity to be pursued. It has been argued in this regard that—on the basis of sweeping away the concept of personal criminal liability, exclusively of a physical nature, by the legislative recognition of such liability as also being of a corporate nature, in antithesis with the legal maxim *societas delinquere non potest*—a kind of “virtual criminal liability” could consequently be hypothesized, i.e., for “virtual subjects,”^14^ in any case acting in an “external world” (Ingarrica, 2022, p. 8), although the identification of “organizational fault,” in truth, seems more pertinent to the system than to the individual.^15^ In addition, from a strictly operational point of view, even in all these disparate cases, one is confronted with the possibility of the user-persons being able to regenerate their “innocence” through simply editing their account (whether in a planned or forced way). Moreover, even with regard to what is being protected, it is not at all certain—according to some—that legal provisions designed for “real and living persons” and not for avatars or software codes (the integrity of which would perhaps protect against acts of mere damage to “things”^16^) can be used, even including cases of violent annihilation fall more into the sphere of violations related to relational speech and expression^17^ (and can then be taken into account as factors in broader hypotheses of threats, persecutory acts and private violence against physically living individuals); and this regardless of the hypothesis of the probability of a virtual dimension of the punishment inflicted (Eberhart, 2022).^18^ Clearly, this brings us back, philosophically speaking, to the dilemma of the recognition of legal (and real) personalities to that of computerized creations/projections (Chalmers, 2022, p. 331 ff.) and also, in another way, to all the ethical questions concerning the determination of inadmissibility and the (s)objectivity gradations of seriousness with respect to the perpetration of unlawful acts in computerized role-playing systems (Luck, 2009).^19^ In any case, what is virtual is by no means, *per se*, fictitious (so much so that the earliest recorded acts of ‘sexual assault’ in the context of reproduced life^20^ left specific post-traumatic traces^21^; Dibbell, 1993; although the very definability of power behaviors enacted as concretizing—socially and not legally—acts of such violence is not only uncertain, with regard to delimitation and content,^22^ but should also be read in the complex light of “real” social constructionism; MacKinnon, 1997).^23^ It should be remembered, however, that the equalization between consideration of “artistic” or digital representation and the essence of physical-anagrammatic characters has already been jurisprudentially made for the purposes of the existence of particular crimes, first and foremost in the area of child pornography (*cf.* Brizi, 2017; Chibelli, 2017), within a framework of social sanctionability,^24^ expanding against what can be treated as an inaccurate “cultural danger” (at the expense of strict factuality), so much so that even self-rejuvenation, traceable in the creation and dissemination of one’s own “winking” images, has been branded as *paedo-baiting* (Stokes, 2021).

Nonetheless, whether we refer to the existence of *digital twins* (El Saddik, 2018), that virtually duplicate the presence and properties of physical counterparts (where the emergent behavior of what is physical is present within a complex system; Grieves and Vickers, 2017), and interact with them, whether we are witnessing digital nativities divorced from other levels,^25^ linked to their own socially normative ecosystems functional to intangible content. In each case, we seem to notice a general formal regulatory deficiency^26^—in some ways, however, sensible in the face of the ontological fragmentariness of the criminal justice system and, perhaps, partly solvable by customary civil law enforcement—and labile terms on the subject of “meta-rights” (Continiello, 2022). It seems obvious how we do not relate here to dynamics internal to mere experimentation with emotional-relational simulators (almost artificial “reduced scales of existence”): it is not a matter of sanctioning actions and attempts by personal “lab-like” characters, but to weigh the actual personal sanctionability of anagraphically determined individuals (or their possible subjection to social-preventive measures, where the commission of a *preater delictum* can be hypothesized).

Moreover, the fact that these appear to be globally shared concerns also raises the question of what kind of oversight and control is feasible in light of the nature and type of political systems of reference (see Shead, 2022), not least because standards of social unacceptability can worryingly undermine the idea of the imperative of compliance with the rule of law.

In essence, the complexity and innovative property of the metaverse—which, first of all, is a social place—derive not so much from its being a tool but, rather, from its being an unbroken proper scope as opposed to “non-virtual.” In addition, in light of this, it should be stressed once more that while the declination of the metaverse has often emphasized it as being a space of opportunity (see Duan et al., 2021) and freedom or, at the opposite end, danger (*cf.* Senno, 2022) and prevarication and oppression, this is precisely due to it being a space in itself, a continuum and a complement, integration of and equal to the classically real.

Some theorists of media studies, even those defined as surreality, might therefore be judged as being useful in gaining a greater understanding of the definitional and regulatory issues underlying the new reality progression.^27^

## Author contributions

The author confirms being the sole contributor of this work and has approved it for publication.

## Footnotes

^1^On the complexity of the prosumption process, particularly in relation to data generated in the cultural sphere, see Beer and Burrows (2013).

^2^It should be noted that the construction of current—and somewhat “elusive”—metaversal dimensionalities is in line with the technological-cultural evolution observed in recent decades. For example, Bell and Kennedy (2007) discuss the deep-rooted implication of cyberculture on various individual and social aspects.

^3^Alternatively, existing offenses are aggravated by the use of new technologies, as in the case of legislation already enacted, for instance, on the subject of terrorism and stalking.

^4^Suffice it to recall, as legally established, cases of incitement to commit a criminal offense or cases involving the solicitation of minors under the age of 16—by means of trickery, flattery, or threats, carried out also through the use of the Internet or other networks or means of communication—for the purpose of committing particularly odious offenses.

^5^In addition to the perception of the actions endured.

^6^Reference is made to the definition of the Internet not as a place or space but as an instrument for multiple forms of digital communication (Pica, 2004, p. 429).

^7^The “challenges” that would be faced—as schematically expressed in Interpol (2022b)—would be recognizing digital identities, protecting data collected in various ways, managing operability between different systems, ensuring cybersecurity, counteracting the effects of the digital divide, and regulating new criminal acts.

^8^After all, the world of *Second Life* was already subject not only to the “external” monitoring of postal police sections against various alleged offenses but also, albeit in a piecemeal fashion, to the establishment of specific divisions operating through virtual agents.

^9^With ramifications, among other things, on the labor law and labor market levels, as well as in the area strictly related to the regulation of international trade.

^10^This, also in relation to debates on the legal capacity and capacity to act—and consequent responsibility—to be accorded, in various capacities, to the composite and non-homogeneous set of “virtual identities.” Specifically, addressing concerns of ethical sovereignty, legal autonomy, and technical independence of “artificial humans” (Budnik and Tedeev, 2023). Pascuzzi discusses the various meanings of digital identity in the cybernetic context (Pascuzzi, 2020, pp. 44 ff.).

^11^The transition from physical to cyber-related crime requires a specific criminological approach (Jaishankar, 2008). Therefore, cyber-criminology has been developed to study the causality of crimes committed in cyberspace and their physical impact (Moise, 2020).

^12^Such as that of total or partial accomplishment of the criminal act (with all the problematic nature of the computerized dematerialization of the action) or of the residence of the person who committed it, for the purpose of identifying the competent court.

^13^Nonetheless, on the problem of determining the place where the crime was committed, when the passive subject is indeterminate, as in relation to cases of danger, especially abstract (Camplani, 2020).

^14^On the tendency to apply a model (of abstraction) based on an idea of anthropization, also in the creation of an “electronic legal personality” (Avila Negri, 2021). In parallel, Magro reflects on the “criminal capacity” of informally artificial creations in the light of causal theory and the finalistic doctrine of action (Magro, 2019, pp. 1203 ff.).

^15^Another, but related, issue is that of the hypothetical legal liability of artificial intelligence systems. On this subject, *amplius* Taddei Elmi and Contaldo (2020). The debate also appears to have practical implications in light of the pervasive organization of the ‘internet of things’ (or “of everythings”).

^16^For a discussion of charges of unauthorized access to and misuse of the data of others (Gentile, 2008). For our legal system, we can, for instance, refer to the incriminating provisions for computer fraud and abusive access to a computer or telecommunications system.

^17^Reading what is enunciated as realizing the action it deals with (and the narrative as a reality in itself) can certainly conceptually bring the field of discourse closer to the field of action, but obviously it cannot lead to any arbitrary dislocation on the normative level.

^18^Clearly, the violation of more or less formalized rules of netiquette, even if only related to acts of griefing, can lead to very different consequences—collectively shared or individually attributed—from the most frequent ostracism to the rarest “avataricide.”

^19^Discussions that lead back to social and juridical rules that are not entirely homogeneous with regards to the criteria that we could define as the “(intermittent) suspension of virtuality,” i.e., the consideration of real disvalue for fictional operations (where thought or being, rather than action, almost seems to be punished).

^20^It should be noted that, although the reproduction-simulation of reality carried out in that context still appeared sketchy in terms of stereoscopic impressions, it nonetheless allowed for the “embodiment” of an emotional transfiguration of the peripersonal space, so to say.

^21^In addition to “para-judicial” requests for “digital civilization,” some are even advocating for “virtual castrations” (Turkle, 1995, p. 251).

^22^The case appears to be even more complex to resolve than that of sexual violence committed at a distance and without physical contact between the offender and victim (which was established as so by the Supreme Court in Judgment No. 25266/2020, III Criminal Section), since the approach between the parties is present but not in physical reality (taken into consideration, however, in the wording of the Supreme Court, as the place where specific acts are performed).

^23^This also pertains to what can be categorized as “nonconsensual porn” acted out in the metaverse (Annison, 2022, p. 41); this label appears to be easy to guess, but the content of which appears at least elusive (between unauthorized use of others’ images and coercion on *avatars*). For some further reference to the debated constitution of new forms of pornographic expression, see Cole and Maiberg (2022) and Pandey (2022); while for some hints at the innovative possibilities of grooming and child molestation, see White (2022) and Park (2022). Moreover, this also leads to necessary reminders of the uneven amalgam that gives concrete form to charges of exploitation and, also, aiding and abetting prostitution.

^24^Which, moreover, can affect the legal interpretations of adjudicable behavior in light of the appeal to social sense employed in reading them.

^25^Perhaps in the context of the “dematerialization” of body and space and the rejection of clear-cut dualism (on which, philosophically and politically, see Haraway, 1991).

^26^On the subject of applicable regulations, the metaverse itself, as a space for action, does not yet appear to be coherently regulated at a European level. Only on the subject of artificial intelligence is there a proposal for a European regulation—COM (2021) 206—capable of establishing harmonized rules.

^27^Given that the *Web* itself, widely considered, has re-proposed the issue of the constant dialectic—not without its highly problematic nature—between technology and rules (see, e.g., Mensi and Falletta, 2021).

## References

[ref1] Accoto C. (2017). Il mondo dato. Cinque brevi lezioni di filosofia digitale. Milan: Egea.

[ref2] Annison T. (2022). The Future of Financial Crime in the Metaverse. Fighting Crypto-crime in Web3.0. https://www.elliptic.co/hubfs/Crime%20in%20the%20Metaverse%202022%20final.pdf.

[ref3] Aterno S. (2023). “Profili penali della vita nel Metaverso” in Metaverso. Diritti degli utenti – piattaforme digitali – privacy – diritto d’autore – profili penali – blockchain e NF. eds. Cassano G. Scorza G. (Pisa: Pacini), 421–430.

[ref4] Avila Negri S. M. C. (2021). Robot as legal person: electronic personhood in robotics and artificial intelligence. Front. Robotics and AI 8:789327. doi: 10.3389/frobt.2021.789327, PMID: 35004866 PMC8734654

[ref5] Ball M. (2022). The Metaverse. And how it will revolutionize everything. New York: Liveright Publishing Corporation.

[ref6] Bassoli E. (2009). La disciplina giuridica della seconda vita in Internet: l’esperienza Second Life. Informatica e diritto 1, 165–189.

[ref7] Beer D. Burrows R. (2013). Popular culture, digital archives and the new social life of data. Theory, Culture & Society 30, 47–71. doi: 10.1177/0263276413476542

[ref8] Bell D. Kennedy B. M. (2007). The Cybercultures reader. London: Routledge.

[ref9] Boyd D. (2010). “Social network sites as networked publics: affordances, dynamics, and implications” in Networked self. Identity, community, and culture on social network sites. ed. Papacharissi Z. (New York: Routledge), 39–58.

[ref10] Brizi L. (2017). La nozione di “pornografia virtuale”: verso un dominio della pericolosità sul fatto? Cassaz. Penal. 11, 4039–4059.

[ref11] Budnik R. Tedeev A. (2023). Initial designs of artificial humans: intellectualproperty and ethical aspects. Law Innov. Technol. 15, 222–240. doi: 10.1080/17579961.2023.2184141

[ref12] Caliandro A. Gandini A. (2017). Qualitative research in digital environments. A research toolkit. New York: Routledge.

[ref13] Camplani F. (2020). Locus commissi delicti, norme di collegamento e reati informatici a soggetto passivo indeterminato. Archivio Penale 2, 1–33.

[ref14] Chalmers D. J. (2022). Reality+. Virtual worlds and the problems of philosophy. New York-London: Norton & Company.

[ref15] Chibelli A. (2017). La Cassazione alle prese con il delitto di pornografia minorile virtuale: lo ‘strano caso’ della pedopornografia a fumetti. Diritto penale contemporaneo 7, 290–298.

[ref16] China Institutes of Contemporary International Relations – CICIR. (2021). Metaverse and National Security Report 2021. Available at: https://mp.weixin.qq.com/s/hlN7k-_4ZSftpyE2qNAGeA.

[ref17] Cole S. Maiberg E. (2022). “They Can’t Stop Us”: People Are Having Sex With 3D Avatars of Their Exes and Celebrities. Available at: https://www.vice.com/en/article/j5yzpk/they-cant-stop-us-people-are-having-sex-with-3d-avatars-of-their-exes-and-celebrities.

[ref18] Continiello A. (2022). Le nuove frontiere del diritto penale nel Metaverso. Elucubrazioni metagiuridiche o problematica reale? Giurisprudenza Penale 5, 1–11.

[ref19] Council of the European Union, General Secretariat – C.EU. (2022). Metaverse - Virtual world, real challenges. Available at: https://www.consilium.europa.eu/media/54987/metaverse-paper-9-march-2022.pdf.

[ref20] De Simone F. (2023). Il *locus commissi delicti* nel metaverso: eterotopia o *non lieu*? De iustitia 3, 81–92.

[ref21] De Vivo M. C. (2009). Viaggio nei metaversi alla ricerca del diritto perduto. Informatica e diritto 1, 191–225.

[ref22] Dertouzos M. (2001). The unfinished revolution. Human-centered computers and what they can do for us. New York: Harper Collins.

[ref23] Dibbell J. (1993). A rape in cyberspace. Village Voice. 51, 36–42.

[ref24] Duan H. Li J. Fan S. Lin Z. Wu X. Cai W. (2021). Metaverse for social good: a university campus prototype. *Proceedings of the 29th ACM International Conference on Multimedia*, 153–161.

[ref25] Durante M. (2019). Potere computazionale. L’impatto delle ICT su diritto, società, sapere. Milan: Meltemi.

[ref26] Eberhart C. (2022). META YOUR MAKER Metaverse experts reveal if you can MURDER someone in virtual world – and whether you can be punished if you’re violent. Available at: https://www.the-sun.com/tech/5066296/can-you-murder-in-metaverse/.

[ref27] El Saddik A. (2018). Digital twins: the convergence of Multimedia technologies. IEEE MultiMedia 25, 87–92. doi: 10.1109/MMUL.2018.023121167

[ref28] Ferri W. (2022). Sul metaverso per ora non c’è nessuno, ma è già arrivata la polizia, Available at: https://www.lindipendente.online/2022/10/27/sul-metaverso-per-ora-non-ce-nessuno-ma-e-gia-arrivata-la-polizia/.

[ref29] Floridi L. (2015). The Onlife manifesto. Being human in a Hyperconnected era. Cham-Heidelberg-New York-Dordrecht-London: Springer.

[ref30] Fornasari G. (2004). “Il ruolo della esigibilità nella definizione della responsabilità penale del provider” in Il diritto penale dell’informatica nell’epoca di Internet. ed. Picotti L. (Padua: Cedam), 423–432.

[ref31] Gentile V. (2008). Uccide un avatar. Arrestata. Available at: https://www.punto-informatico.it/uccide-un-avatar-arrestata/.

[ref32] Grieves M. Vickers J. (2017). “Digital twin: mitigating unpredictable, undesirable emergent behavior in complex systems” in Transdisciplinary perspectives on complex systems. eds. Kahlen F.-J. Flummerfelt S. Alves A. (Switzerland: Springer), 85–113.

[ref33] Haraway D. J. (1991). “A cyborg manifesto: science, technology, and socialist-feminism in the late twentieth century” in Simians, cyborgs and women. The reinvention of nature, Ead (New York: Routledge), 149–181.

[ref34] Ingarrica D. (2022). Metaverso criminale. Quali interazioni nel presente nazionale e quali sfide globali del prossimo futuro. Giurisprudenza Penale 9, 1–18.

[ref35] Interpol (2022a). INTERPOL lance le premier métavers policier mondial, Available at: https://www.interpol.int/fr/Actualites-et-evenements/Actualites/2022/INTERPOL-launches-first-global-police-Metaverse.

[ref36] Interpol (2022b). Interpol Technology Assessment. Report on Metaverse. Available at: https://www.interpol.int/content/download/18440/file/INTERPOL%20Tech%20Assessment-%20Metaverse.pdf.

[ref37] Jaishankar K. (2008). “Space transition theory of cyber crimes” in Crimes of the internet. eds. Schmallager F. Pittaro M. (Upper Saddle River: Prentice Hall), 283–301.

[ref38] Kostenko O. V. (2022). Electronic jurisdiction, Metaverse, artificial intelligence, digital personality, digital avatar, neural networks: theory, practice, perspective. World Sci 1, 1–13. doi: 10.31435/rsglobal_ws/30012022/7751

[ref39] Lastowka F. G. Hunter D. (2004). The Laws of the virtual worlds. Calif. Law Rev. 92, 1–73. doi: 10.2307/3481444

[ref40] Lombard M. Ditton T. (1997). At the heart of it all: the concept of presence. J. Comput.-Mediat. Commun. 3:2. doi: 10.1111/j.1083-6101.1997.tb00072.x

[ref41] Lombardi A. (2022). “Aggredita sessualmente nel Metaverso”. La denuncia di una ricercatrice fa scattare la reazione di Meta: “Distanza di sicurezza tra gli avatar”. Available at: https://www.repubblica.it/esteri/2022/02/14/news/metaverso-337711044/.

[ref42] Luck M. (2009). The gamer’s dilemma: an analysis of the arguments for the moral distinction between virtual murder and virtual paedophilia. Ethics Inf. Technol. 11, 31–36. doi: 10.1007/s10676-008-9168-4

[ref43] Lupton D. (2015). Digital sociology. London-New York: Routledge.

[ref44] MacKinnon R. (1997). Virtual Rape. J. Comput.-Mediat. Commun. 2:4. doi: 10.1111/j.1083-6101.1997.tb00200.x

[ref45] Magro M. B. (2019). “Robot, cyborg *e intelligenze artificiali*” in Cybercrime. eds. Cadoppi A. Canestrari S. Manna A. Papa M. (Milan: Utet), 1179–1212.

[ref46] Marres N. (2017). Digital sociology. The reinvention of social research. London: Wiley.

[ref47] Martellozzo E. Jane E. (2019). Cybercrime and its victims. Oxford-New York: Routledge.

[ref48] Mazer (2022). Crime in the Metaverse. Available at: https://mazerspace.com/crime-in-the-metaverse/.

[ref49] McDonnell N. Wildman N. (2019). Virtual reality: digital or fictional? Disputatio 11, 371–397. doi: 10.2478/disp-2019-0004

[ref50] Mensi M. Falletta P. (2021). Il diritto del web. Casi e materiali. Padua: Cedam.

[ref51] Moise A. C. (2020). Cyber-criminology – a new field of scientific research and criminological investigation. J. Law Admin. Sci. 14, 121–126.

[ref52] Morozov E. (2011). The net delusion. How not to liberate the world. London: Allen Lane.

[ref53] Oleksy T. Wnuk A. Piskorska M. (2022). Migration to the metaverse and its predictors: attachment to virtual places and metaverse-related threat. Comput. Hum. Behav. 141, 1–10. doi: 10.1016/j.chb.2022.107642

[ref54] Orton-Johnson K. Prior N., ed. (2023). The Palgrave Macmillan digital sociology. Critical perspectives. Basingstoke: Palgrave Macmillan.

[ref55] Pandey K. (2022). Should We Ban Porn In the Metaverse?. Available at: https://www.jumpstartmag.com/should-we-ban-porn-in-the-metaverse/?mc_cid=444d34c45e&mc_

[ref56] Park D. (2022). South Korea struggles to prevent sexual harassment of minors in the metaverse. Available at: https://forkast.news/south-korea-sexual-harassment-minors-metaverse/.

[ref57] Pascali M. (2008). Fenomenologia delle “videoviolenze”. Commissione, rappresentazione e creazione della violenza. Naples: Giannini.

[ref58] Pascali M. (2022). The “activity of choice” on the web and the production of intellectual property rights: the media consumption between free service and unpaid work. Труды по Интеллектуальной Собственности / Works on Intellectual Property 40, 70–72. doi: 10.17323/tis.2022.14221

[ref59] Pascali M. (2023). Derive socio-relazionali e patologie giuridiche nell’era digitale. Parolechiave 9, 63–87.

[ref60] Pascuzzi G. (2020). Il diritto dell’era digitale. Bologna: Il Mulino.

[ref61] Patel N. J. (2021). Reality or Fiction?. Available at: https://medium.com/kabuni/fiction-vs-non-fiction-98aa0098f3b0.

[ref62] Pica G. (2004). “Internet”, in Digesto delle discipline penalistiche (Milan: Utet).

[ref63] Pierattini L. (2022). Il metaverso ha già un problema enorme con le molestie sessuali e per questo Meta è corsa ai ripari. Available at: https://www.gqitalia.it/tech/article/metaverso-molestie-sessuali-confine-personale.

[ref64] Riva G. (2014). Nativi digitali. Crescere e apprendere nel mondo dei nuovi media. Bologna: Il Mulino.

[ref65] Rogers R. (2013). Digital methods. Cambridge: MIT.

[ref66] Rolfes L. J. (2022). The Legitimacy of Rules of Virtual Communities. Available at: https://edoc.hu-berlin.de/bitstream/handle/18452/24607/dissertation_rolfes_louis.pdf?sequence=5.

[ref67] Scorza G. (2022). In principio era Internet e lo immaginavamo diverso. Rivista italiana di informatica e diritto 4, 13–16.

[ref68] Senno S. (2022). Il Metaverso, un’opportunità per la società o per il terrorismo? Sfide nell’ambito della prevenzione. Available at: https://www.amistades.info/post/metaverso-opportunita-societa-o-per-terrorismo.

[ref69] Shead S. (2022). Serious crime in the metaverse should be outlawed by the U.N., UAE minister says. Available at: https://www.cnbc.com/2022/05/25/metaverse-murders-need-to-be-policed-says-uae-tech-minister.html.

[ref70] Stephenson N. (1992). Snow crash. New York: Bantam.

[ref71] Stokes R. (2021). Una creator di OnlyFans che modifica le sue foto è accusata di ‘paedo baiting’. Available at: https://www.vice.com/it/article/k78x3e/coconutkitty143-accuse-foto-modificate.

[ref72] Suler J. (2004). The online disinhibition effect. CyberPsycol. Behav. 7, 321–326. doi: 10.1089/1094931041291295 15257832

[ref73] Taddei Elmi G. Contaldo A., ed. (2020). Algoritmi giuridici. Ius condendum o “fantadiritto”?. Pisa: Pacini.

[ref74] Torrão F. (2014). “Direito Penal, Globalização e Pós-modernidade (desconstrução do paradigma liberal)”, in Multiculturalismo e Direito Penal, ed. Pizarro Beleza T. Caeiro P. Lacerda F. de da Costa Pinto (Coimbra: Almedina), 59–96.

[ref75] Turkle S. (1995). Life on the screen. Identity in the age of the internet. New York: Simon and Schuster.

[ref76] Vanorio F. (2021). Metaverso e Sicurezza nazionale. Internet 3.0 e nuovo ordine mondiale digitale. Available at: https://www.strategicstudies.it/wp-content/uploads/2021/12/Edizioni-Machiavelli-Metaverso-e-Sicurezza-Nazionale.pdf.

[ref77] von Uexküll J. (2013). Ambienti animali ed ambienti umani. Macerata: Quodlibet.

[ref78] Weatherby L. (2018). Delete Your Account: On the Theory of Platform Capitalism. Available at: https://lareviewofbooks.org/article/delete-your-account-on-the-theory-of-platform-capitalism/.

[ref79] White L. (2022). Facebook’s Metaverse is already a hot spot for child predators, claim experts. Available at: https://stealthoptional.com/metaverse/facebooks-metaverse-child-predators/.

[ref80] Wildman N. McDonnell N. (2020). The puzzle of virtual theft. Analysis 80, 493–499. doi: 10.1093/analys/anaa005

[ref81] Yee N. Bailenson J. N. Ducheneaut N. (2009). The Proteus effect: implications of transformed digital self-representation on online and offline behavior. Commun. Res. 36, 285–312. doi: 10.1177/0093650208330254

